# Single-stage hybrid repair of a ruptured Kommerell diverticulum associated with dextrorotation, bovine arch, and bicuspid aortic valve

**DOI:** 10.1186/s13019-019-0942-7

**Published:** 2019-06-26

**Authors:** Jeko M. Madjarov, Michael G. Katz, Sarah M. Gubara, Svetozar Madzharov, Kevin Reames, Sophia Madjarova, Francis Robicsek

**Affiliations:** 1 Department of Cardiovascular Surgery, Sanger Heart & Vascular Institute, Charlotte, NC USA; 2 Department of Cardiology, Cardiovascular Research Center, Icahn School of Medicine at Mount Sinai, 1470 Madison Ave, Box 1030, New York, NY 10029-6574 USA

**Keywords:** Kommerell diverticulum, Congenital heart anomalies, Hybrid surgical treatment

## Abstract

**Background:**

A strategy for the surgical repair of ruptured Kommerell diverticulum has not yet been established. The aim of this study is to demonstrate that this entity could be associated with a number of other cardiac anomalies and this lesion can be successfully treated by a hybrid approach.

**Case presentation:**

The patient, with a combination of ruptured Kommerell diverticulum, dextrorotation, bovine arch, and bicuspid aortic valve, underwent emergency surgery. A single stage hybrid surgical/endovascular repair including subclavian artery revascularization, aortic resection with open proximal anastomosis under circulatory arrest, endovascular stenting, and valve repair was performed. Histological studies indicated the presence of the aortic wall media degeneration. Postoperative course was uneventful and patient is free of symptoms during 2-year follow up.

**Conclusions:**

Less invasive hybrid technique is safe and effective treatment option. Accumulated knowledge of Kommerell diverticulum has lead to understanding the best clinical treatment for this complicated aortic anomaly.

## Background

Kommerell diverticulum (KD) is a congenital anomaly arising from an embryologic remnant of the dorsal fourth aortic arch. It can occur with right and left aortic arch configurations and aberrant origin of both subclavian arteries [1]. The natural history of KD is not known and studies are limited to small series. Aortic morphology is often challenging in this pathology. KD is usually asymptomatic and in the absence of symptoms is considered to be a benign anatomic variant. However, this diverticulum can cause serious complications, which include distal embolization, aneurysm formation with compression of adjacent structures, aortic dissection, and spontaneous rupture. Although indications for elective operative treatment of patients with KD continue to be debated, the presence of KD is a risk factor of an acute aortic syndrome and therefore should be an indication for intervention. The conventional surgical technique includes open repair with resection and replacement of the involved segment of the aorta with an interposition graft. However, during the last decade surgical options have expanded to include endovascular and hybrid approaches [2]. This report describes a successful single-stage hybrid repair of a patient with a ruptured Kommerell diverticulum with dextrorotation, bovine arch and a bicuspid aortic valve with insufficiency.

## Case presentation

**Fig. 1:**
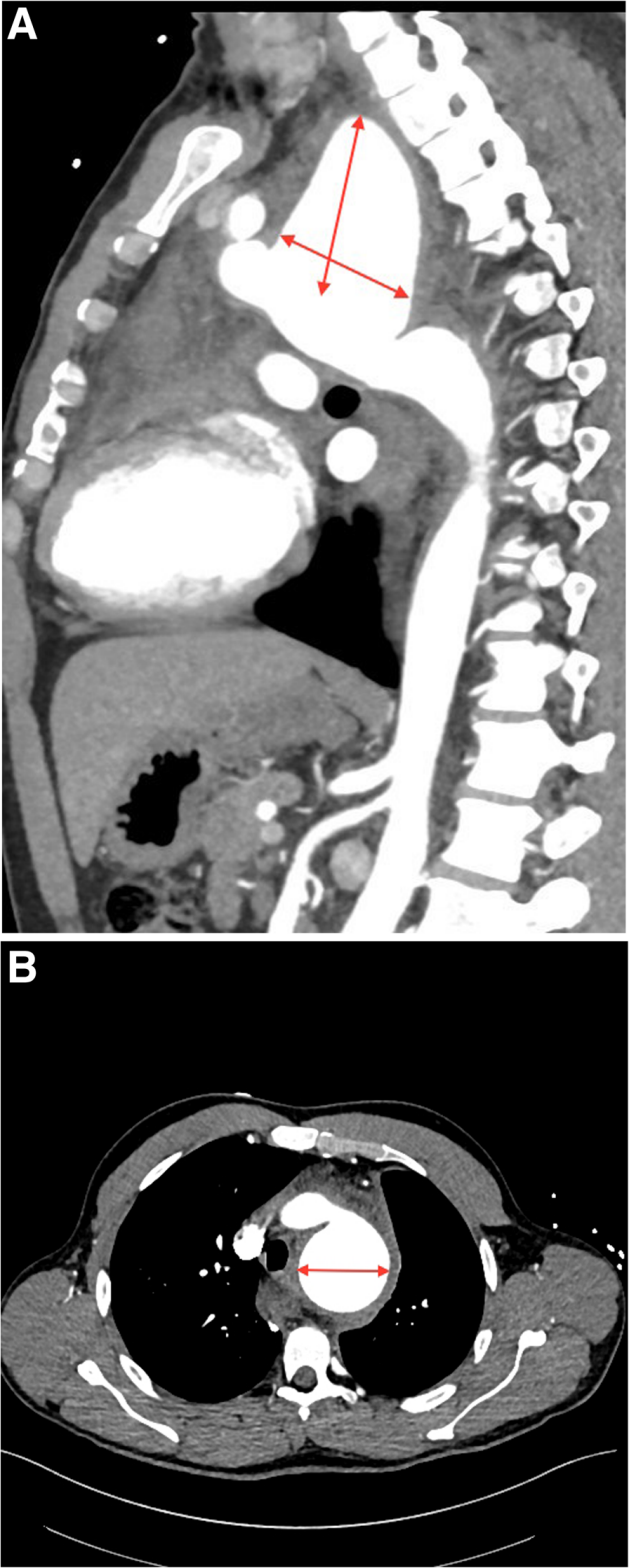
**a** Sagittal contract-enhanced CT-scan reconstruction demonstrated an 8.5-cm saccular aneurysm in the distal aortic arch. **b** Axial contract-enhanced CT-scan showing transversal diameter of Kommerell diverticulum with surrounding mediastinal hematoma suggesting diverticulum rupture

A 44-year-old male was admitted to the emergency department with sudden onset of severe mid-sternal chest pain radiating to the back. He did not report complaints of dysphagia lusoria or hoarseness. The patient was hemodynamically stable and the physical examination was unremarkable. Laboratory results showed no significant deviations. The patient has previously been followed for a bicuspid aortic valve, with yearly cardiac echocardiography. At the time of his current presentation a contrast-enhanced computer tomography (CT) scan revealed a contained rupture of a saccular aneurysm of the base of the left subclavian artery - 7 × 8.5 cm in diameter. The aneurysm extended to the transverse aortic arch with evidence of a large diffuse mediastinal hematoma, small left pleural effusion and bovine aortic arch (Fig. 1a, b). Cardiac echocardiography demonstrated a bicuspid aortic valve with moderate aortic regurgitation grade 2+: jet width 40% of LV outflow tract, regurgitate fraction 35%, end-systolic dimension 50 mm, end-diastolic dimension 65 mm, end-diastolic volume 150 ml/m^2^, end-systolic volume 55 ml/m^2^, LV EF 50%.. After reviewing the radiographic studies, the patient was urgently taken to the operating room.

### Surgical technique

The right axillary artery, left common carotid artery, left subclavian artery and left common femoral artery were exposed. Right auxiliary artery “chimney graft” was created using 8 mm Terumo (Terumo, Vascutek, Ann Arbor, MI) graft in an end-to-side fashion via right subclavicular incision. Next, left common carotid artery-left subclavian artery bypass was performed using an 8–mm Terumo straight graft with end-to-side (to the carotid artery) and end-to-end anastomosis (to the left subclavian artery). The left subclavian artery was completely divided, and proximal part was oversewn.

A median sternotomy was performed, great vessels were dissected, and the anatomy was confirmed. The area of contained rupture was distal to the origin of KD, originating at the base of the left subclavian artery and measuring 8.5 cm in maximum diameter. Heparin was introduced per weight protocol and the patient was placed on cardiopulmonary bypass with arterial access via the right axillary artery conduit and venous access in the right atrium. Systemic cooling was undertaken to 28 °C. Seldiger’s technique was utilized to gain percutaneous access via the left common femoral artery (CFA). This allowed us to use intravascular ultrasound and interrogate from the aortic root to the left CFA, confirming the distal aortic arch aneurysm and intact bovine aortic arch. The diameter of the ascending aorta was 38 mm and native descending aorta was 24 mm. Due to the lack of adequate “landing zone” proximally, the acuity of the situation and the patient’s age purely endovascular approach was not considered. After clamping at the base of the innominate artery under conditions of moderate hypothermia circulatory arrest (28 C), antegrade cerebral perfusion via the chimney graft was initiated.

After initiating circulatory arrest, the aorta was transected at the proximal level of the bovine arch. The bovine arch was detached from the arch and the stump was over sewn with 4–0 Prolene. This provided us with a reliable ~ 3 cm of landing zone. Over the previously placed “through-and-through” wire (Landerquist wire, Cook Medical, Bloomington, IN) from the left common femoral artery access to the aortic arch, we delivered the first endograft (Medtronic 28x28x150 mm, Minneapolis, MN), starting immediately at our newly created landing zone across the Kommerell diverticulum and into the healthy descending aorta. Next we sutured a 26 mm Terumo graft (with 14 mm side branch) to the proximal aortic arch. The Bovine arch was re-anastomosed to the 14 mm Terumo side branch, using 4–0 Prolene in end-to-end fashion. This was done in a way to provide 5 cm of distance from the debranched Bovine arch to the proximal extent of the first piece of endograft. Using the same “through-and-through” wire, we delivered the second piece endograft (28x28x150 mm, WL Gore Inc., Flagstaff, AZ), starting just distal to the debranched bovine arch, across through the previous arch anastomosis and into the first piece endograft, completing our hydrid arch repair, providing two layers of endograft material at the level of the transverse arch and across the base of the aneurysm.

**Fig. 2:**
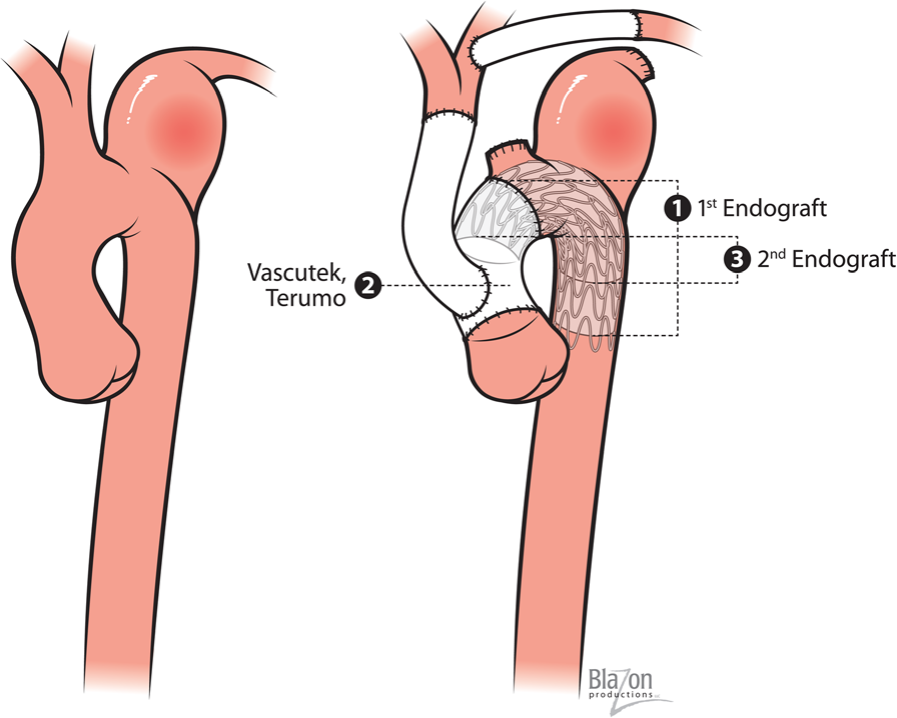
Single stage hybrid repair of ruptured Kommerell diverticulum with first and second endograft

An overlap of 4 cm between the Gore endograft and the 26 mm Terumo graft was secured. At least 8 cm of overlap was also assured between the two separate endografts. After de-airing, aortic clamp was applied proximal to the debranching 14 mm Terumo graft. Perfusion to the distal body with systemic rewarming was initiated. The ascending aorta was excised at the level of the sinotubular junction and send to pathology. The bicuspid aortic valve was reconstructed using subcommissural annuloplasty technique performed with 4–0 Prolene pledgeted felt sutures. A second piece of 26 mm Terumo graft was used to replace the ascending aorta. The anastomosis at the sinotubular junction was created in end-to-end manner using 4–0 Prolene suture. After adequate tailoring, required because of the cardiac dextrorotation with abnormal position for the aortic root (very deep in the posterior mediastinum), the neo-ascending aorta was anastomosed to the neo arch with 4–0 Prolene suture in a running fashion (Fig. 2). After de-airing, the aortic cross clamp was removed. The patient was subsequently weaned off from cardiopulmonary bypass without difficulties. Time of circulatory arrest was 28 min. Cross-Clamp time was 95 min and cardio-pulmonary bypass time was 170 min. For cardioprotection, we utilized Del Nido solution delivered in retrograde fashion through the coronary sinus.

We did not use blood transfusion or pro-coagulants intraoperatively. No aortic insufficiency was revealed on postoperative transesophageal echocardiography. The flow velocity in the both vertebral arteries was normal. Due to apparent osteopenia, longitudinal, rigid sternal fixation was undertaken.

Postoperative course was uneventful and the patient was discharged on postoperative day 5.

**Fig. 3:**
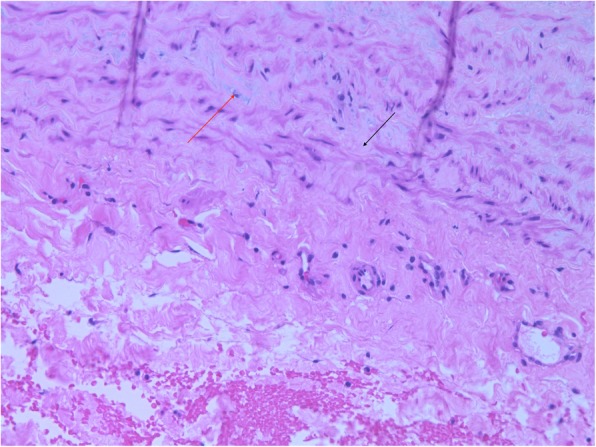
Aortic wall segment with medial degeneration, depletion of smooth muscle cells (black arrow) and deposition of mucoid material (red arrow) (hematoxylin-eosin, original magnification × 100)

Histologic assessment of resected ascending aorta revealed mucoid medial degeneration with fibrosis, and lipid deposition (Fig. 3). Three- and eighteen-month CT-scans have shown no evidence of endoleak with normal perfusion of all the arch vessels. On the last CTA, we also observed positive aortic remodeling with distal arch aneurysm which was decreased in size from 7 × 8.5 cm to 3 × 3.5 cm.

## Discussion and conclusions

### Associated congenital anomalies

Congenital aortic arch malformations present a large spectrum of variations and the prevalence of these abnormalities varies between 1 and 2% among the general population. Kommerell diverticulum is one of such aortic arch anomalies and is defined as an aortic diverticulum arising at the origin of a subclavian artery from the proximal descending aorta. Although the anatomical variants of this anomaly are quite well described in the literature, we found only isolated reports concerning associated congenital anomalies [3, 4]. To the best of our knowledge there is no information regarding the combination of KD with dextrorotation, bovine arch, and bicuspid aortic valve and subsequent single stage surgical treatment.

*Dextrorotation* is a morphologic entity characterized by heart rotation to the.

right in both frontal and transverse planes of the body, while the location and function of the cardiac chambers are safe and normal. In contrast to dextrocardia, this anomaly is not associated with situs inversus, but is frequently complicated by additional cardiac malformations [5]. Due to the dextrorotation of the heart, the aorta was much deeper and more posterior in the mediastinum, whereas the pulmonary artery was anterior and rotated to the right. This presented us with additional technical challenges, requiring “special tailoring” in the acute settings of our repair.

*Bovine arch* represents the most common variation of the aortic arch. In the past, it was considered as a physiological abnormality with no clinical impact but now much more attention is being given. The presence of bovine arch could pose significant challenges to the cardiac surgeon with influence on outcome. Moreover, currently the existence of bovine arch is considered a risk factor for inflammatory aortic disease [6].

*Bicuspid aortic valve* (BAV) is the most common congenital cardiac defect. BAV is often associated with other congenital lesions. Despite its importance, our understanding of BAV disease is incomplete. Patients with this anomaly may develop aortic post-stenotic dilatation and BAV can exaggerate genetic abnormalities of the aortic wall. Based on our previous observations we believe that with a bicuspid aortic valve paired with another form of aortopathy, such as coarctation, Kommerell diverticulum, and bovine arch, the ascending aorta is at higher risk of pathological aortic wall degeneration [7].

During the last decade, there has been increasing appreciation for the role of an underlying aortic disease associated with the diverticulum rupture [8]. In histological specimens, we observed a significant degeneration of the aortic media. The presence of large amounts of acidic mucinous polysaccharides in the aortic media accompanied by loss and fragmentation of elastic fibers, can lead to weakness of the aortic wall, which is susceptible to rupture.

The presence of KD may predispose towards development of aortic aneurysm, aortic dissection, and rupture. Up to 6% of KD’s patients have been reported to present with rupture and 53% with either rupture or dissection [9]. There is a strong correlation between the KD rupture and mortality rate. A few decades ago, regardless of treatment, mortality after KD spontaneous rupture was 100% [1]. In recent years, with the advent of new methods of surgical management, this statistic has improved. Nevertheless, KD rupture with mediastinal hemorrhage or hematoma is a life-threatening emergency and requires immediate surgical intervention.

### Treatment options

A strategy for surgical treatment of Kommerell diverticulum has not yet been finally established. This is most likely because the choice of surgical treatment, which is usually based on the individual anatomy of the patient, is very diverse. A number of surgical techniques with or without use of partial or total cardiopulmonary bypass, hypothermia, and circulatory arrest have been proposed for treatment of these aneurysms [2, 8–10]. The conventional treatment for the malformation includes complete removal of the KD, with reconstruction of the subclavian artery (SA), graft replacement of the aorta with SA ligation or reconstruction, endoaneurysmorrhaphy with SA reconstruction, total arch replacement with reconstruction of SA, and endovascular repair in selected patients. Perfusion strategies are also varied and include left heart or total cardiopulmonary bypass, moderate hypothermia, and deep hypothermic circulatory arrest with arterial cannulation in the femoral artery or descending aorta and venous cannulation in the pulmonary vein or common femoral vein.

In our patient, the KD originated in close proximity to the bovine arch, making proximal control and cross-clamping challenging. Therefore we used circulatory arrest and constructed open distal anastomosis without the impediment of a clamp. Due to dextrorotation we had to use a second piece of Terumo graft to connect the sinotubular junction to our neo-arch graft because of the severely angulated aorta. Our patient had no procedure-related complications or recurrence of symptoms. We believe that the use of this technique has facilitated the conduction of the procedure and improved its efficacy and safety. As we continue to master our strategy in treating complex aortic pathology like Kommerell diverticulum, the additional knowledge and understanding of this cardiovascular pathology will be quite valuable in determining the timing, surgical technique and extent of aortic reconstruction.

We also want to underscore that with the advancement of our surgical technique armamentarium and the improvements of medical technology, conditions that were previously considered “rare” but associated with adverse outcomes should be evaluated and addressed accordingly. In fact, many centers of excellence have started seeing these rare pathologies more frequently. Thus, it’s important that we have a better understanding of these pathologies and strategies for managing these patients, possibly providing excellent and long lasting results. We believe that there is an opportunity to collaborate with medical technology to come up with these new strategies.

In our case, we had a bicuspid aortic valve with ascending aortic dilatation, combined with Bovine aortic arch and Kommerell diverticulum. The cardiac dextrorotation made the “tailoring” of the grafts a little more challenging. With better medical technological designs, we should be able to address these sometimes rather complex aortic cases, even when done in an elective manner.

## Data Availability

Not applicable.

## Abbreviations

BAV: Bicuspid aortic valve
CT: Computed tomography
KD: Kommerell diverticulum
SA: Subclavian artery
